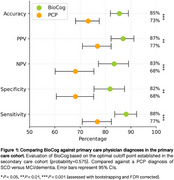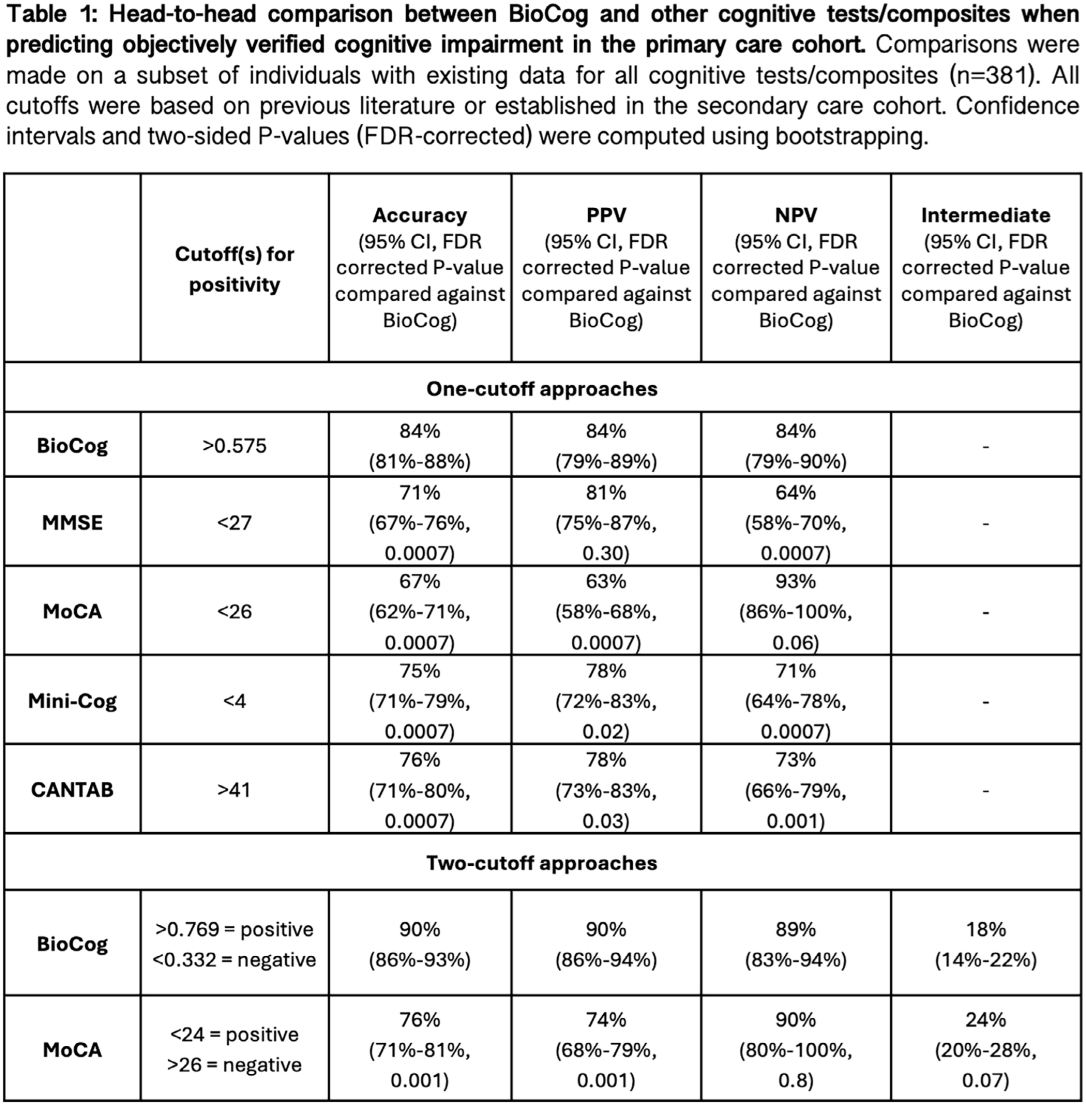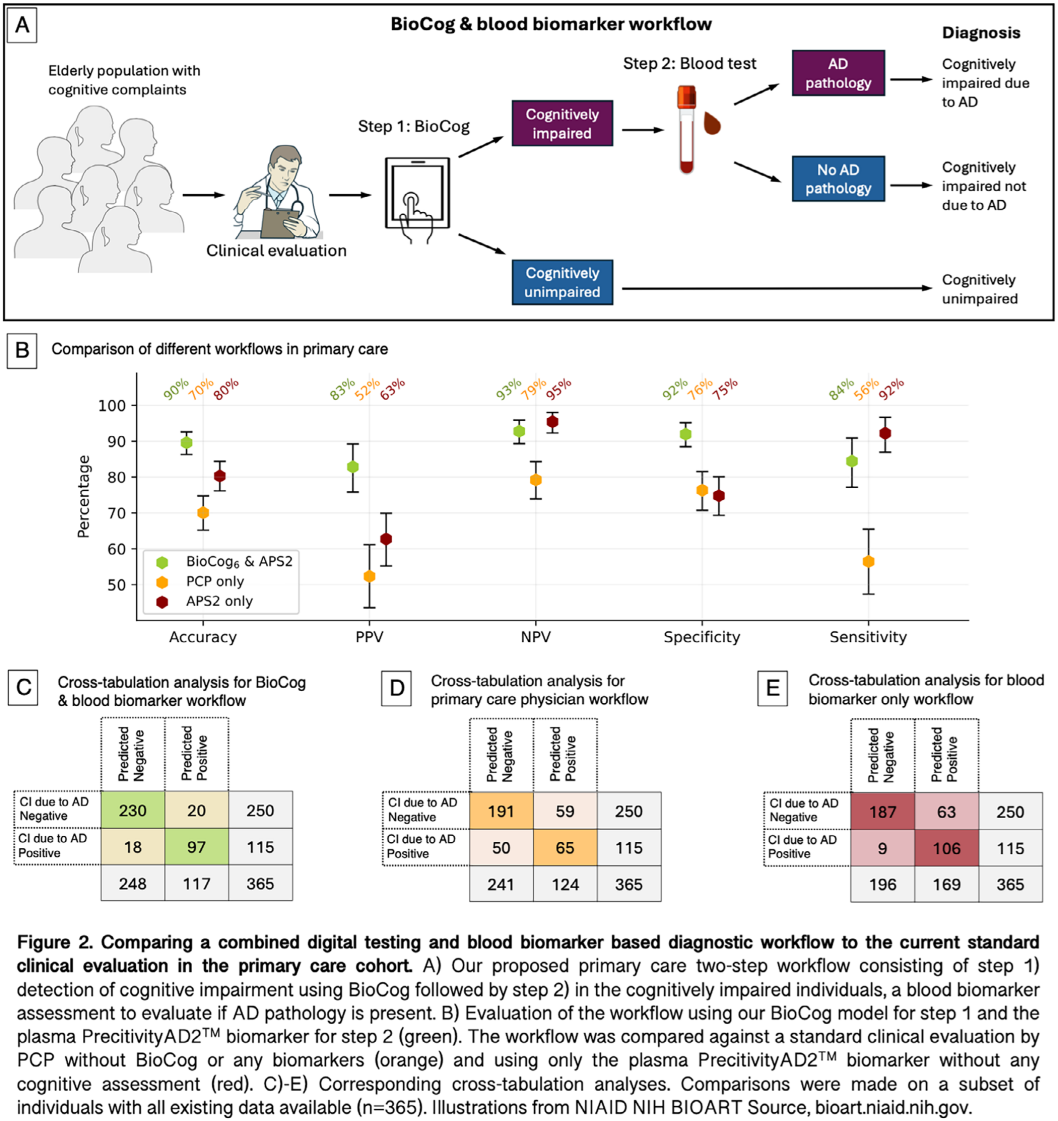# A self‐administered digital cognitive test together with a blood test accurately detect clinical Alzheimer's disease in primary care

**DOI:** 10.1002/alz70857_104975

**Published:** 2025-12-25

**Authors:** Pontus Tideman, Linda Karlsson, Olof Strandberg, Susanna Calling, Ruben Smith, Patrik Midlöv, Philip B. Verghese, Joel B. Braunstein, Niklas Mattsson‐Carlgren, Erik Stomrud, Sebastian Palmqvist, Oskar Hansson

**Affiliations:** ^1^ Clinical Memory Research Unit, Lund University, Lund, Sweden; ^2^ Memory Clinic, Skåne University Hospital, Malmö, Skåne, Sweden; ^3^ Clinical Memory Research Unit, Department of Clinical Sciences, Lund University, Lund, Sweden; ^4^ Clinical Memory Research Unit, Department of Clinical Sciences Malmö, Lund University, Lund, Sweden; ^5^ Center for Primary Health Care Research, Department of Clinical Sciences, Lund University, Malmö, Sweden; ^6^ Clinical Memory Research Unit, Lund University, Malmö, Skåne, Sweden; ^7^ C2N Diagnostics LLC, St. Louis, MO, USA; ^8^ C2N Diagnostics, LLC, St. Louis, MO, USA

## Abstract

**Background:**

After the development of disease‐modifying amyloid‐β targeting therapies for patients with cognitive impairment due to Alzheimer's disease (AD), there is an urgent need to efficiently detect this patient population. However, when these patients first enter the health care system in primary care, there is often a lack of time and expertise to conduct assisted, in‐clinic, cognitive testing. Additionally, the administration and interpretation of cognitive tests vary among primary care providers, both within and between countries. Therefore, we created and evaluated a brief and self‐administered digital cognitive battery (BioCog) as a stand‐alone test and combined with blood biomarkers.

**Methods:**

BioCog takes 10‐15 minutes to perform, assessing memory, processing speed and orientation. Based on its sub‐scores, we developed a logistic regression model and established cutoffs in a Swedish memory clinic cohort (*n* = 223). The model and cutoffs were validated in an independent Swedish primary care cohort comprising of 19 primary care centers (*n* = 403, mean [SD] age 77 [8.0] years and 48% were male). All participants had cognitive symptoms that the responsible physician wanted to investigate further. The primary outcome was objectively verified cognitive impairment, established using the Repeatable Battery for the Assessment of Neuropsychological Status (RBANS).

**Results:**

In primary care, BioCog had an accuracy of 85% (95% CI, 81‐89%) when using a single cutoff to predict cognitive impairment, which was significantly better than the assessment of primary care physicians (accuracy 73%, 95% CI, 68‐77%; Figure 1). The accuracy increased to 90% when using two cutoffs. BioCog had significantly higher accuracy than standard paper‐and‐pencil tests (i.e., MMSE, MoCA, Mini‐Cog) and another digital cognitive test (CANTAB), Table 1. Furthermore, BioCog combined with an accurate blood test (PrecitivityAD2^TM^) could detect clinically symptomatic and biomarker‐verified AD with an accuracy of 90% (95% CI, 86%‐92%), significantly better compared to standard clinical evaluation without BioCog and blood biomarkers (accuracy 70%, 95% CI, 65%‐74%), or when using the blood test alone (accuracy 80%, 95% CI, 76%‐84%), Figure 2.

**Conclusions:**

A brief, newly developed self‐administered digital cognitive test battery (BioCog) can detect cognitive impairment and can, when combined with a blood test, accurately identify clinical AD in primary care.